# 

**Published:** 1916-11-25

**Authors:** 


### Hospital and Institutional Results in 1915.

**Glamorgan County Lunatic Asylum.**—There were 1,913 patients on the asylum register at the close of the year, and the medical superintendent reports that the institution is greatly overcrowded. The recovery rate, calculated upon the total number of admissions, is 29.8. Four children were born in the asylum; one of the mothers suffers from recurrent mania, was a patient before her marriage, and her mother has been in the asylum for many years.

**Inverness District Asylum.**—There were 714 persons on the asylum register at the end of the statistical year, May 15, 1916. The death-rate was 12.8 per cent., the average for district asylums in Scotland being 11 per cent. The male patients have continued to perform, under the care and guidance of the attendants, a large amount of useful work in the grounds, including road-making and excavations for drains.

**Melrose District Asylum.**—This asylum, the erection and furnishing of which cost £174,000, is administered under the Roxburgh District Board of Control. The average number of patients during 1915 was 419, of whom 87 were temporarily accommodated by arrangement with the Edinburgh and Glasgow District Asylums.

**Royal Hospital for Incurables, Putney Heath.**—The financial year of this institution ended on September 30, 1916. The income for those twelve months was £35,854 (including £10,804 legacies) and the expenditure £35,600. The only source of income that has notably increased is under the heading of "Life subscriptions," which are really donations with certain voting privileges. The ordinary expenditure is £2,000 higher than in the previous twelve months. The total sum raised by a "festival luncheon" at the Hall of the Grocers' Company in June last was £9,350.

### Gifts to Hospitals.

Under the will of Mr. Edward Brewis, a retired stock and share dealer, of Winchelsea Road, Tottenham, the London Hospital receives a bequest of £5,000.

Mr. and Mrs. F. W. Sykes, of Lindley, who have always been warm supporters of the Huddersfield Royal Infirmary, have sent it a cheque for £1,000 for current accounts.

To support Sir John Ellerman's offer of £1,000 to the British Red Cross Society if nine other persons would give a like amount, £1,000 has been received from Sir James Roberts, of Saltaire, and Mr. T. Fagelund respectively.

Birmingham charities will benefit by the distribution of the residue of the estate of Mr. Alfred Knight, of Acock's Green, to the Birmingham and Midland Skin Hospital, the Eye Hospital, the Queen's Hospital, and the Royal Orthopædic Hospital.

In memory of his only son, Lieutenant John Bury, 5th East Lancashire R.F.A. (T.F.), who was killed in Gallipoli, Mr. A. S. Bury, of Arden Hall, Accrington, has given £500 to the Accrington and District Victoria Hospital for the endowment of a cot in the children's ward.

### Passing Events and Topics.

### National Health Fair.

A National Health Fair will be held from December 4 to 9 at the Institute of Hygiene, Devonshire Street, W., for the promotion of first-aid, sick-nursing, invalid cooking, and mothercraft. The programme is an interesting one, and includes a series of "ten-minute health talks."

### Royal Hospital for Incurables.

Invitations have been issued for the Founder's Day Celebration to be held at the Royal Hospital for Incurables, Putney Heath, on November 28, at 7 p.m. The wards may be visited, there will be "a little music," and Mr. T. W. Wickham, the chairman of the board, and Mr. Eliot Read, the grandson of the founder, will briefly address the guests.

### Federated Malay States Military Hospital.

The military hospital maintained at Blackmore End, Kimpton (Herts), by public subscriptions from the Federated Malay States, for sick and wounded soldiers, has been enlarged by the addition of a new ward containing forty-four beds. This brings the total number of beds available to over 200. A large recreation-room is in course of construction.

### Amateur Prescribing.

Having been advised by a friend, as a cure for rheumatism, to bathe in water in which calcium carbide had been dissolved, a Brooklyn lamplighter, says the New York *Medical Record*, procured a can of this, filled his bath-tub, seated himself in it, and then emptied the contents of the can into the water. As a consequence there was an explosion and a flash of flame, and the ardent experimenter, instead of being cured of rheumatism, added severe burns to his other troubles.

### War Shrines and Maternity Centres.

The credit of a new departure in war shrines is due to the St. Anne's School of Mothercraft, Clerkenwell. On November 3 the Bishop of Stepney unveiled one to the memory of the husbands and sons of those connected with the school. The shrine has been made and designed by Mr. Eagle, whose wife is secretary of the school. The design is simple and dignified, with a cross in the middle of it and a panel on each side—one for those now serving and one for those already fallen; the name of the late founder of the school, Mr. M. Lloyd Baker, heading the list.

### ST. THOMAS'S HOSPITAL.

House Appointments.—The following have been selected as house officers from November 14, 1916: *Casualty Officers and Resident Anæsthetists.*—C. N. Carter, B.A. Cantab., M.R.C.S., L.R.C.P.; W. S. Brown, B.A. Cantab., M.R.C.S., L.R.C.P.; C. Y. Roberts, M.R.C.S., L.R.C.P.; W. T. Beswick, B.A. Cantab. *Resident House Physicians.*—G. W. J. Bousfield; A. Mavrogordato, M.A. Oxon., M.R.C.S., L.R.C.P.; J. M. Wall, L.M.S.S.A.; G. M. Kendall, B.A. Cantab., M.R.C.S., L.R.C.P. *Resident House Surgeons.*—N. S. Nairne, M.R.C.S., L.R.C.P.; W. Marriott; J. S. White, M.B., B.Ch., N.U. Ireland; P. Sai, M.R.C.S., L.R.C.P. *House Surgeon to Block 8.*—O. H. Hyman. *Obstetric House Physician.*—K. E. Crompton, B.A., M.B., B.C. Cantab. *Clinical Assistant (Children's Medical).*—J. R. Harris.

* The Hospital, November 11, p. 112.

### MR. PETT RIDGE'S APPEAL.

The Royal Free Hospital Appeal Committee has enlisted the powerful pen of Mr. W. Pett Ridge in its extension scheme effort. The story of the hospital's work and the tragic moral of the wasted child-life of to-day is graphically told by Mr. Pett Ridge in a brochure, "The Missing Million: An Army that might have been," which is being distributed broadcast.

### Matrons' and Sisters' Appointments.

Aberdare and District General Hospital.—Miss Frances Hood has been appointed matron. She was trained at Manchester Royal Infirmary, where she was later staff nurse and day sister. She has also been staff nurse and undertaken matron's duties at Rawcliffe Hospital, Chorley, theatre sister and assistant superintendent nurse at the Anlaby Road Infirmary, Hull, and superintendent nurse at Coventry Union Infirmary.

Exeter Isolation Hospital.—Miss Bredha Curtin Leahy has been appointed matron. She was trained at the Royal Devon and Exeter Hospital, where she was later sister, and has also been staff nurse at the North-Eastern Fever Hospital, Tottenham.

Sheffield, Jessop Hospital.—Miss Louisa Helen Balton has been appointed matron. She was trained at University College Hospital, and has since been night superintendent, ward sister, and deputy-matron of the Samaritan Free Hospital for Women, and matron of Tewkesbury General Hospital. She also holds the certificate of the Central Midwives Board.

Denby and Cumberworth Hospital for Soldiers, Denby Dale.—Miss M. A. Meadows has been appointed nurse-matron. She was trained at Chester Royal Infirmary and at City Hospital East, Liverpool, and has been sister at Bradford Children's Hospital, matron of Parkgate Convalescent Home, and night sister at Leasowe Children's Hospital, Cheshire.

Lincoln County Hospital.—Miss Flora Heslington has been appointed sister of a military ward. She was trained at Monkwearmouth Hospital, Sunderland, and has since been charge nurse at Tynemouth Jubilee Infirmary, and sister at the Cameron Hospital, West Hartlepool, and the Royal Bath Hospital, Harrogate.

**NOTE.—Particulars of Appointments for insertion in this column will be welcomed.**

For the College of Nursing and State Registration, see p. 169.

### CONTENTS.

RATES OF SUBSCRIPTION: Twelve months, 6/6; Six months, 4/-; Three months, 2/-; post free to any address in the United Kingdom. Abroad, Twelve months, 8/8; Six months, 5/-; Three months, 2/6, post free. THE HOSPITAL "Index" to Volume LX., April to September 1916, is now ready, price 2½d. per copy, post free. Address The Manager, "THE HOSPITAL," The Hospital Building, 28/29 Southampton Street, Strand, London, W.C.

